# Combination of Resting State fMRI, DTI, and sMRI Data to Discriminate Schizophrenia by *N*-way MCCA + jICA

**DOI:** 10.3389/fnhum.2013.00235

**Published:** 2013-05-29

**Authors:** Jing Sui, Hao He, Qingbao Yu, Jiayu Chen, Jack Rogers, Godfrey D. Pearlson, Andrew Mayer, Juan Bustillo, Jose Canive, Vince D. Calhoun

**Affiliations:** ^1^The Mind Research Network, Lovelace Biomedical and Environmental Research Institute, Albuquerque, NM, USA; ^2^LIAMA Center for Computational Medicine, National Laboratory of Pattern Recognition, Institute of Automation, Chinese Academy of Sciences, Beijing, China; ^3^Department of ECE, University of New Mexico, Albuquerque, NM, USA; ^4^Division of Natural Science, New College of Florida, Sarasota, FL, USA; ^5^Olin Neuropsychiatry Research Center, Hartford, CT, USA; ^6^Department of Psychiatry, Yale University, New Haven, CT, USA; ^7^Department of Neurobiology, Yale University, New Haven, CT, USA; ^8^Department of Psychiatry, University of New Mexico, Albuquerque, NM, USA; ^9^Department of Neuroscience, University of New Mexico, Albuquerque, NM, USA; ^10^Psychiatry Research Program, New Mexico VA Health Care System, Albuquerque, NM, USA

**Keywords:** multimodal fusion, mCCA + jICA, resting state fMRI, DTI, sMRI, schizophrenia, ALFF, GM

## Abstract

Multimodal brain imaging data have shown increasing utility in answering both scientifically interesting and clinically relevant questions. Each brain imaging technique provides a different view of brain function or structure, while multimodal fusion capitalizes on the strength of each and may uncover hidden relationships that can merge findings from separate neuroimaging studies. However, most current approaches have focused on pair-wise fusion and there is still relatively little work on *N*-way data fusion and examination of the relationships among multiple data types. We recently developed an approach called “mCCA + jICA” as a novel multi-way fusion method which is able to investigate the disease risk factors that are either shared or distinct across multiple modalities as well as the full correspondence across modalities. In this paper, we applied this model to combine resting state fMRI (amplitude of low-frequency fluctuation, ALFF), gray matter (GM) density, and DTI (fractional anisotropy, FA) data, in order to elucidate the abnormalities underlying schizophrenia patients (SZs, *n* = 35) relative to healthy controls (HCs, *n* = 28). Both modality-common and modality-unique abnormal regions were identified in SZs, which were then used for successful classification for seven modality-combinations, showing the potential for a broad applicability of the mCCA + jICA model and its results. In addition, a pair of GM-DTI components showed significant correlation with the positive symptom subscale of Positive and Negative Syndrome Scale (PANSS), suggesting that GM density changes in default model network along with white-matter disruption in anterior thalamic radiation are associated with increased positive PANSS. Findings suggest the DTI anisotropy changes in frontal lobe may relate to the corresponding functional/structural changes in prefrontal cortex and superior temporal gyrus that are thought to play a role in the clinical expression of SZ.

## Introduction

Multimodal brain imaging techniques are playing increasingly important roles in elucidating structural and functional properties in normal and diseased brains, as well as providing the conceptual glue to bind together data from multiple types or levels of analysis. The related computational methods are also valuable for clinical research on the mechanisms of disease progression. The goal of multimodal fusion is to capitalize on the strength of each imaging modality as well as their inter-relationships in a joint analysis, rather than to analyze separately.

Each imaging modality provides a different view of brain function or structure, and data fusion capitalizes on the strengths of each imaging modality/task and their inter-relationships in a joint analysis, creating an important tool to help unravel the black box of psychotic disorders, such as schizophrenia (SZ) (Calhoun et al., 2006; Sui et al., 2012a). Recent advances in data fusion include integrating multiple (task) fMRI data sets (Sui et al., 2009b, 2010; Kim et al., 2010) from the same participant to specify common versus specific sources of activity to a greater degree than traditional general linear model-based approaches. This can increase confidence when making conclusions about the functional significance of brain regions and activation changes in brain diseases. In addition, the combination of function and structure may provide more informative insights into both altered brain patterns and connectivity (McCarley et al., 2008; Michael et al., 2010; Sui et al., 2011). For example, a lower and different function–structure connection is often found in patients with SZs compared with healthy controls (HCs) (Zhou et al., 2008; Venkataraman et al., 2010; Camchong et al., 2011; Michael et al., 2011), while varied brain patterns are also identified frequently (Calhoun et al., 2008; Xu et al., 2009; Brown et al., 2012; Lu et al., 2012).

### Why go beyond two modalities?

However, most current approaches have focused on pair-wise fusion and there is still relatively little work on *N*-way data fusion and examination of the full relationships among multiple data types. Given the availability of more powerful MR scanners, there are typically more than two imaging modalities available for one participant. Hence, we believe the joint multivariate analysis of multiple data types (e.g., resting state fMRI, task-related fMRI, DTI, and sMRI) will improve our ability to understand brain diseases. We have proposed an *N*-way fusion model, “multi-set canonical correlation analysis (mCCA) + joint independent component analysis,” i.e., “mCCA + jICA,” which successfully identified both modal-common and modal-unique group-discriminative patterns for HCs and SZs via combination of task-related fMRI, DTI, and sMRI data (Sui et al., 2013). Considering the importance of the interpretation of multi-way features, the method and tool we propose will enable examination of full correspondence across *N* modalities by achieving reliable inter-modality associations and high decomposition accuracy together, thus making discoveries of changes in one modality causing related alterations in distant, but connected regions in other modalities possible.

To our knowledge, there have been only a few reports combining three or more types of brain imaging data to investigate brain disorders (e.g., Correa et al., 2009) examined changes that are related across fMRI, sMRI, and EEG data for SZ (Groves et al., 2011) compared Alzheimer’s patients and age-matched controls by combining gray matter (GM) density and three diffusion data measures [fractional anisotropy (FA), mean diffusivity, and tensor mode]. For resting state fMRI data, several pair-wise fusion applications have been reported (Teipel et al., 2010; Long et al., 2012; Segall et al., 2012); however, there has been no report that combine resting state fMRI with other two or more different types of brain imaging data to study SZ.

In this project, we applied the *N-*way fusion model, “mCCA + jICA” (Sui et al., 2013), to compare not only modality-common but also modality-unique abnormalities among resting state fMRI, sMRI, and DTI data, which is the first attempt to combine such three types of data to discriminate SZ patients (*n* = 35) from HCs (*n* = 28). *N*-way fusion of brain imaging data is more challenging than pair-wise combination, since many fusion applications rely on studying correlations between highly distilled measures (e.g., small regions of interest), while there is still relatively little examination of the full relationships among data types. The method and tools we propose will enable such an examination and can be potentially useful for identification of unique biomarkers of brain disorders. Furthermore, the high-dimensional neuroimaging data is typically very noisy and massive redundancy reduction is usually necessary to facilitate the identification of relationships among modalities. For this purpose, each modality is first reduced to a “feature” for each subject, which tends to be more tractable than working with the large-scale original data (Calhoun and Adali, 2009) and provides a simpler space to link the data (Smith et al., 2009), e.g., an fMRI contrast map from the general linear model, a GM segmentation image from the sMRI scan and voxel-wise DTI measures such as FA. For resting state fMRI data, we used the amplitude of low-frequency fluctuation (ALFF) as fusion input (Zang et al., 2007; Zou et al., 2008; Calhoun and Allen, 2013), which has been used previously for default mode or other applications in multiple papers (Calhoun et al., 2012; Turner et al., 2012; Yu et al., 2012b, 2013).

## Materials and Methods

### Theory development

Existing multivariate fusion methods have different optimization priorities and limitations: some enable common as well as distinct levels of connection among modalities, such as mCCA (Correa et al., 2009) and partial least squares (PLS) (Lin et al., 2003; Chen et al., 2009), but their separated sources may not be sufficiently spatially sparse. For example, mCCA maximizes the inter-subject covariation across two sets of features and generates two linked variables, one from each dataset, i.e., canonical variants (CVs); which correlate with each other only on the same indices (rows) and their corresponding correlation values are called canonical correlation coefficients (CCC). This strategy allows for both common and distinct aspects of two features, but the brain maps of several components may look similar when the CCCs are not sufficiently distinct. Some approaches perform well in spatial decomposition, such as jICA (Calhoun et al., 2006) and linked ICA (Groves et al., 2012), which aim at maximizing the independence among estimated sources combining more than two modalities, but only allow a common mixing matrix. These two methods enable detection of features common to all modalities at the expense of features which may be distinct to one or more of them (a situation which becomes more likely when combining more than two modalities). Multiple previous studies that combined function and structure (Olesen et al., 2003; Rykhlevskaia et al., 2008; Camara et al., 2010; Sui et al., 2012b) provide support for the assumption that components decomposed from each modality have some degree of correlation between their mixing profiles among subjects. This motivates our data-driven model that is optimized for both flexibility in inter-modal associations and high capability on source separation.

**Figure 1:**
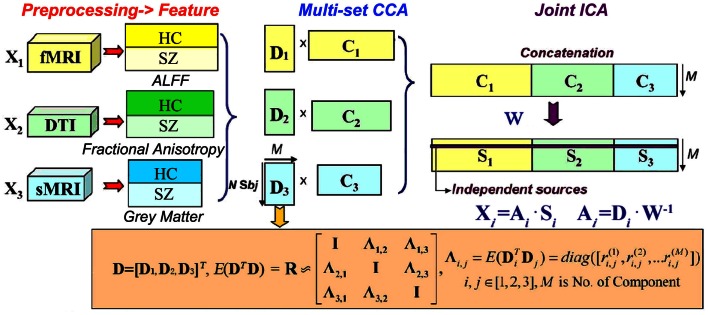
***N*-way mCCA + jICA fusion strategy of for real human data (*n* = 3 in this study)**.

The basic strategy of mCCA + jICA is shown in Figure 1. MCCA is first adopted to project the data in a space so that the correlations among mixing profiles (**D***_k_*, *k* = 1, 2, …, *n*) of *n* (*n* = 3 in this study) modalities are jointly maximized (in their sum of squared correlations). The resulting CVs **D***_k_* are sorted by correlation which provides a closer initial match to the potential highly or weakly correlated mixing profiles between components, which will make the subsequent application of jICA more reliable. At this time, the associated maps **C***_k_* may not be completely separated by mCCA. We then apply jICA on the concatenated maps (**C**_1,_ **C**_2,…,_ **C***_n_*) to obtain the final maximally independent source **S***_k_*. In other words, mCCA first relates multiple datasets with flexible linkages (correlation) in their mixing matrices, which matches well with the assumptions of jICA that is subsequently applied to the joint spatial maps. Hence, mCCA and jICA are complementary to one another, and can relax the limitations of each listed above if used together, generating both highly and weakly correlated joint components that are independent.

We assume that the multimodal dataset **X***_k_*, is a linear mixture of *M_k_* sources given by **S***_k_*, mixed with a non-singular mixing matrix (or loading parameters) **A***_k_* for each, *k* denotes modality.

(1) $$X_{k}=A_{k}S_{k}\;k=1,2,\ldots ,n$$

where **X***_k_* is a subjects-by-voxels feature matrix (we use voxels for our description but it could also be, e.g., time points or genes). The sources **S***_k_*, are distinct within each dataset, while the columns of **A***_i_* and **A***_j_* have higher correlation only on their corresponding indices, *i*, *j* ∈ {1, 2, …, *n*} *i* ≠ *j* are modality number. Given that there are *N* subjects, typically, the number of voxels *L* in **X***_k_* is much larger than *N*. Due to the high dimensionality and high noise levels in the brain imaging data, order selection is critical to avoid over fitting the data. Using the improved minimum description length (MDL) criterion (Li et al., 2007), the number of independent components *M_k_* are estimated for each modality and we set the final component number for jICA as *M* = max(*M*_1_, *M*_2_, …, *M_n_*). Dimension reduction is then performed on **X***_k_* using singular value decomposition to determine the signal subspace given by

(2) $$Y_{k}=X_{k}E_{k}\;k=1,2,\ldots ,n$$

where **Y***_k_* is in size of *N* × *M* and **E***_k_* contains eigenvectors corresponding to significant (the top *M* highest) singular values. Multi-set CCA (Li et al., 2009) is thus performed on **Y***_k_*, generating the CVs **D***_k_* = **Y***_k_***w***_k_* by maximizing the sum-of-squares of all correlation values in the corresponding columns of **D***_k_* so that

(3) $$E\left\{D_{k}^{T}D_{k}\right\}=I;\;E\left\{D_{k}^{T}D_{j}\right\}\approx \mathrm{diag}\left(r_{k,j}^{\left(1\right)},r_{k,j}^{\left(2\right)}...r_{k,j}^{\left(M\right)}\right)$$

where *k*, *j* ∈ {1, 2, …, *n*}, *k* ≠ *j*. Based on the linear mixture model, we simultaneously obtain the associated components **C***_k_* via **X***_k_* = **D***_k_*⋅**C***_k_*, **C***_k_* = pinv(**D***_k_*)⋅**X***_k_*. However, the performance of mCCA for blind source separation (BSS) may suffer when $r_{k,j}^{\left(1\right)},r_{k,j}^{\left(2\right)}...r_{k,j}^{\left(M\right)}$ are very close in values, which might occur in applications using real brain data, since the multimodal connection among components usually are not very high and could be similar in value (Sui et al., 2011). Therefore, **C***_k_* will typically be a set of sources that are not completely independent. Joint ICA is then implemented on the concatenated maps (**C**_1_, **C**_2,…,_ **C***_n_*), to maximize the independence among joint components by reducing their second and higher order statistical dependencies, as in Eq. (4). ICA as a central tool for BSS has been studied extensively and we utilized Infomax (Bell and Sejnowski, 1995) in our work due to its good stability.

(4) $$\left[S_{1},S_{2}...S_{n}\right]=W\cdot \left[C_{1},C_{2}...C_{n}\right]$$

Finally, *n* sets of independent components **S***_k_* are achieved, with their corresponding mixing matrices **A***_k_* linked via correlation. The proposed scheme “mCCA + jICA” can be summarized as shown in Figure 1.

(5) $$X_{k}=\left(D_{k}\cdot W^{-1}\right)\cdot S_{k},A_{k}=D_{k}\cdot W^{-1}$$

Multi-set canonical correlation analysis + jICA was compared with its alternatives in simulation in Sui et al. (2013), where results show that combination of mCCA and jICA mitigates the performance deficits of each and achieves more reliable and better separation on both sources and mixing matrices. Interestingly, when the estimated component number is higher than the ground truth, the source estimation performance continues to be high, while the estimation of mixing coefficients achieves best performance when *M* equals to true values.

### Human brain data

#### Participants

Multi-set canonical correlation analysis + jICA was applied to DTI, resting state fMRI, and sMRI data of 63 subjects recruited as part of a multimodal SZ center for biomedical research excellence (COBRE) study at the Mind Research Network^1^. Informed consent was obtained from all subjects according to institutional guidelines required by the Institutional Review Board at the University of New Mexico (UNM). Table 1 lists the demographic information. All subjects were screened and excluded if they had history of neurological disorder, history of mental retardation, history of severe head trauma with more than 5 min loss of consciousness, or history of substance abuse, or dependence within the last 12 months (except for nicotine). HCs were free from any Axis I disorder, as assessed with the SCID-NP (Structured Clinical Interview for DSM-IV-TR, Non-patient version). Patients met criteria for SZ defined by the DSM-IV-TR based on the SCID-P interview (First et al., 1995). All patients were on stable medication prior to the fMRI scan session. The two groups did not differ with regard to age, gender, and ethnicity, see Table 1. Symptom scores were determined based on the positive and negative syndrome scale (PANSS) (Kay et al., 1987).

**Table 1:** **Demographic information of the subjects**.

	Num	Age	Gender	Ethnicity
HC	28	39 ± 15	21M/7F	21 Whites
SZ	35	36 ± 12	26M/9F	22 Whites
*p* Value		0.36	0.99	0.58

#### Imaging parameters

All the data were collected on a 3-T Siemens Trio scanner with a 12-channel radio frequency coil at the Mind Research Network. The imaging parameters were as follows: *fMRI*: resting state data were collected with single-shot full *k*-space echo-planar imaging (EPI) with ramp sampling correction using the inter commissural line (AC/PC) (anterior commissure/posterior commissure) as a reference (TR = 2 s, TE = 29 ms, matrix size = 64 × 64, flip angle = 75 °, slice thickness = 3.5 mm, slice gap = 1.05 mm, field of view (FOV) 240 mm, matrix size = 64 × 64, voxel size = 3.75 mm × 3.75 mm × 4.55 mm. *sMRI*: a multi-echo MPRAGE sequence was used with the following parameters: TR/TE/TI = 2530/(1.64, 3.5, 5.36, 7.22, 9.08)/900 ms, flip angle = 7 °, FOV = 256 × 256 mm, slab thickness = 176 mm, matrix size = 256 × 256 × 176, Voxel size = 1 mm × 1 mm × 1 mm, Pixel bandwidth = 650 Hz, Total scan time = 6 min. *DTI*: data was collected along the AC/PC line, throughout the whole brain, FOV = 256 × 256 mm, slice thickness = 2 mm, NEX (number of excitations) = 1, TE = 84 ms, TR = 9,000 ms. A multiple channel radio frequency coil was used, with GRAPPA (generalized autocalibrating partially parallel acquisition) (×2), 30 gradient directions with a diffusion sensitivity, *b* = 800 s/mm^2^. The *b* = 0 experiment was repeated five times, and equally inter-spread between the 30 gradient directions. All *b* = 0 images were registered to the first *b* = 0 image with a six degrees-of-freedom transformation. This was followed by registering the *b* = 800 s/mm^2^ image to the *b* = 0 image immediately before it by an affine 12 degrees-of-freedom transformation. The two transformations were multiplied and then one transformation applied to the *b* = 800 s/mm^2^ image to align it to the first *b* = 0 image. This resulted in all images being registered to the first *b* = 0 image. FLIRT (FMRIB’s Linear Image Registration Tool) was used for all registration steps.

#### Resting state fMRI

Resting-state scans were a minimum of 5 min, 4 s in duration (152 volumes). Subjects were instructed to keep their eyes open during the scan and stare passively at a foveally presented fixation cross, as this is suggested to facilitate network delineation compared to eyes-closed conditions and helps ensure that subjects are awake.

#### fMRI preprocessing

SPM8 software package^2^ was employed to perform fMRI preprocessing. Slice timing was performed with the middle slice as the reference frame. Images were realigned using INRIalign, a motion correction algorithm that is unbiased by local signal changes (Freire et al., 2002). Data were then spatially normalized into the standard Montreal Neurological Institute (MNI) space (Friston et al., 1995) with affine transformation followed by a non-linear approach with 4 × 5 × 4 basis functions. Images (originally collected at 3.75 mm × 3.75 mm × 4.55 mm) were then slightly upsampled to 3 mm × 3 mm × 3 mm, resulting in a data cube of 53 × 63 × 46 voxels. Before smoothing, we further regress out the six motion parameters for each slice to remove the motion effect. Finally, data were spatially smoothed with a Gaussian kernel of full-width half maximum (FWHM) of 10 mm × 10 mm × 10 mm. For the rest fMRI, we extracted the voxel-wise ALFF to generate a map for each subject. The ALFF calculation consisted of computing the fast Fourier transform (FFT) of each voxel time series, taking the square root of the power spectrum to obtain amplitude, and averaging amplitude in (0.01, 0.1) Hz. Prior to computing ALFF, the original 4D fMRI data sets were divided by their global mean (over time and space) to normalize differences in scan intensity units. ALFF maps computed in this manner were used previously in a comparative classification analysis (Erhardt et al., 2011) and the use of ALFF maps in a “second-level” ICA has been previously studied (Calhoun and Allen, 2013).

#### DTI preprocessing

DTI data were preprocessed by FMRIB Software Library (FSL)^3^ and consisted of the following steps: (a) quality check, any gradient directions with excessive motion or vibration artifacts were identified and removed; (b) motion and eddy current correction; (c) correction of gradient directions for any image rotation done during the previous motion correction step; (d) calculation of diffusion tensor and scalar measures such as FA, which were then smoothed and resized to a final 53 × 63 × 46 matrix for each subject, see more details in Sui et al. (2011).

#### sMRI preprocessing

sMRI data were also preprocessed using the SPM8 software package which was used to segment the brain into white-matter (WM), GM, and cerebral spinal fluid with unmodulated normalized parameters via the unified segmentation method (Ashburner and Friston, 2005). After segmentation, the GM images were smoothed to a FWHM Gaussian kernel of 10 mm (White et al., 2001) and re-sliced to a matrix of 53 × 63 × 46 voxels. Subject outlier detection was further performed using a spatial Pearson correlation with the template image, to ensure that all subjects were properly segmented (for details, see Segall et al., 2009).

#### Normalization

After feature extraction (preprocessing), the 3D brain images of each subject were reshaped into a one-dimensional vector and stacked, forming a matrix with dimensions of 63 × number of voxels for each of the three modalities. These three feature matrices were then normalized to have the same average sum-of-squares (computed across all subjects and all voxels/locus for each modality) to ensure all modalities had the same ranges. Following normalization, the relative scaling (a normalization factor) within a given data type was preserved (i.e., 1.08, 0.24, 0.39 for ALFF, FA, GM respectively), but the normalized input units have the same voxel-wise mean square variance for all modalities. Next, the data was processed via the pipeline shown in Figure 1, i.e., dimension reduction → multi-set CCA → jICA → component analysis. The component number was estimated using modified MDL (Li et al., 2007) to be 10, 5, 8 for fMRI, DTI, and sMRI respectively. We thus choose *M* = 10 for the following analysis since we have found that a slight overestimation of the component number does not adversely affect the results in simulation (Sui et al., 2011). Note that the estimated IC number is lower than that used for 4D fMRI data typically, since mCCA + jICA works on extracted features of interests, instead of the original imaging data. However, a considerable amount of variance is retained for the *M* = 10 case, i.e., 95, 96, 99% for fMRI, DTI, and sMRI respectively.

### Analyzing generated components and mixing coefficients

After applying the mCCA + jICA to the human brain data, independent component **S***_k_* and the mixing matrices **A***_k_* for each modality (*k* = 3 in this study) were generated, providing a variety of ways to analyze the inter-correlation between modalities as well as the group differences, as in Sui et al. (2011). In this paper, we are most interested in:

#### Shared/distinct abnormalities

Two-sample *t*-tests were performed on mixing coefficients of each IC for each modality (i.e., first 28 elements corresponding HC versus last 35 elements corresponding SZ from *m*th column of **A***_k_* for the *m*th IC of modality *k*), the results tell us which components are significantly abnormal in SZ. If the components of the same index show group differences in more than one modality, they are called modality-common (or joint) group-discriminative ICs. By contrast, if the component shows significant group difference only in a single modality, it is called a modality-unique group-discriminative IC. That are what we call shared or distinct abnormalities.

#### Inter-modality correlation

We also looked into the column-wise correlations between A_1_, A_2_, and A_3_ pair wisely. It is likely that the joint group-discriminative components have a strong inter-modality correlation between their mixing coefficents, which indicates the interaction and correspondence among modalities.

#### Impact of clinical measures

The derived mixing coefficients also provide a way to investigate the relationships between the identified components and subjects’ clinical data, e.g., the correlation between mixing coefficients of patients for each component and antipsychotic medication doses [standardized as olanzapine equivalents (Gardner et al., 2010)] or PANSS scores. In this paper, we computed the correlation with PANSS (Kay et al., 1987), which rate the scale of severity of positive, negative, and general symptoms in SZ.

#### Potential use for classification

To test the potential use of the identified group-discriminative components (i.e., corresponding rows of **S***_k_* of modality *k*), we next used them to generate features (e.g., the *Z* map above certain threshold) and train a classifier, to see whether they are able to predict diagnosis or serve as potential biomarkers, which may prove the great significance for multimodal analysis.

For each modality, we transferred the group-discriminating components (for ALFF and GM, we use only two ICs with minimum *p* values) into *Z* values and thresholded at |*Z*| > 3.5, generating a mask from each component. The masks of the same modality were then combined and applied to the raw input matrix of each modality, which served as the input to the further classification based on uni-modal and multimodal features. Each individual was assigned one of two class memberships (SZ versus HC). We trained four different classification algorithms: linear support vector machine (LSVM) (Cortes and Vapnik, 1995), radial basis function support vector machine (RSVM) (Amari and Wu, 1999), *k*-nearest neighbor algorithm (KNN) (Geva and Sitte, 1991), and Gaussian naïve bayes (GNB) (McCallum and Nigam, 1998). Each algorithm was trained on 50% of the data (randomly chosen samples) with 10-fold cross validation, and tested on the other half for 1000 times, with the mean and maximal success rate recorded. Because this paper is not mainly focused on classification, we will not address the details of each algorithm. One limitation of this experiment is that the data set used to identify group-discriminating components is the same as the one which we did classification with, since we don’t have other similar resting fMRI-DTI-sMRI data at hand for cross validation and our main aim is to test whether mCCA + jICA is able to serve as an effective feature selection method for group prediction.

## Results

### Group differences in human brain data

**Figure 2:**
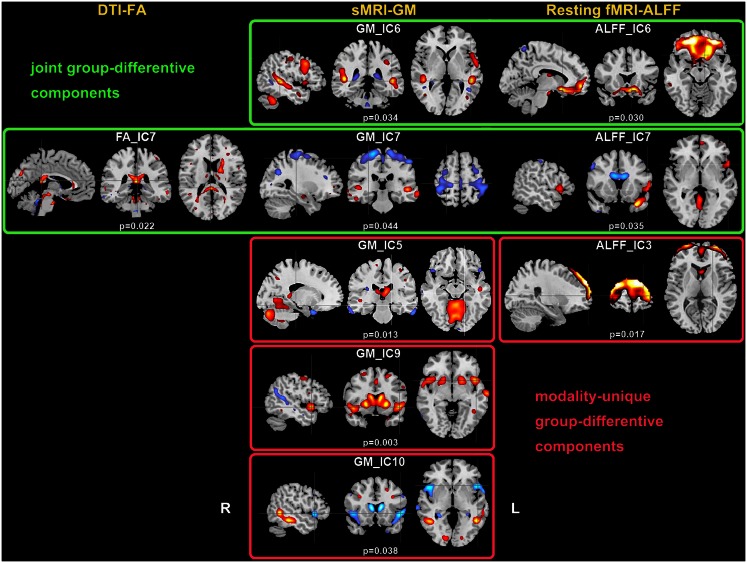
**Group-discriminating regions across three modalities, with a threshold of |*Z*| > 2.5**. Two-sample *t*-tests were performed on mixing coefficients of each IC for each modality. If the components of the same index show group differences in more than one modality, they are called modality-common (or joint) group-discriminative ICs in green frames; otherwise, it is called a modality-unique group-discriminative IC, e.g., GM_IC5, ALFF_IC3 in red frames.

Two-sample *t*-tests found both modality-common group-discriminative ICs (e.g., IC6 and IC7 in green frames, as shown in Figure 2) as well as modality-unique group-discriminative ICs, e.g., GM_IC5, ALFF_IC3 in our case. Interestingly, the modal-connection between joint-discriminative ICs indicate significant correlations (GM-ALFF IC6: *r* = 0.28, *p* = 0.025; FA-GM IC7: *r* = 0.38, *p* = 0.002; FA-ALFF IC7: *r* = 0.31, *p* = 0.015) between their mixing profiles.

### Correlation with PANSS scores

**Figure 3:**
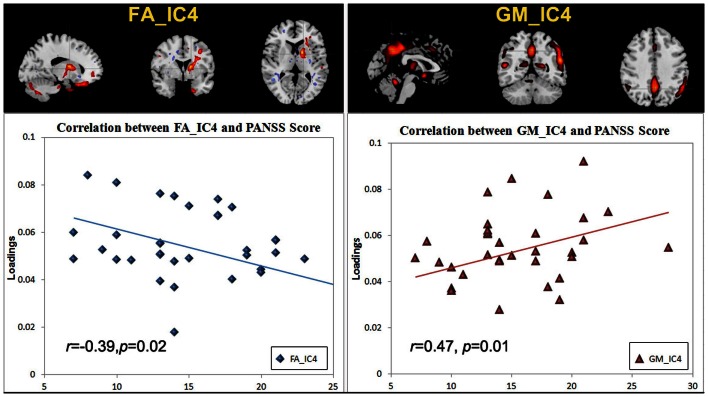
**The scatter plots and linear trends of components with significant correlation between positive PANSS score and its loadings**.

There was no significant correlation regarding the antipsychotic medication doses. However, two ICs: FA_IC4 (anterior thalamic radiation, ATR and superior longitudinal fasciculus, SLF) and GM_IC4 (subregions of the default mode) were significantly correlated with positive PANSS scores, while there was no significant correlation with negative PANSS score. The scatter plots and linear trends are shown in Figure 3.

The specific identified regions of the components of interest and their abbreviations are summarized in Table 2 for resting state fMRI components (Talairach labels), Table 3 for DTI (WM tracts), and Table 4 for sMRI (MNI labels) respectively. For fMRI and sMRI, each IC is transformed into a *Z* map by dividing its standard deviation across all voxels, and the voxels above the threshold (|*Z*| > 2.5) were converted from MNI coordinates to Talairach coordinates and entered into a database to provide anatomic and functional labels for the right (R) and left (L) hemispheres. The volume of identified voxels in each area is provided in cubic centimeters (cm^3^). Within each area, the maximum *Z* value and its MNI coordinates are provided for all three tables. To summarize the WM results, we used the Johns Hopkins WM tractography atlas (from FSL) (Hua et al., 2008), from which 20 structures were identified; mostly large bundles. In Table 3, the WM tract labels, the identified volume (cc), and the percentage that indicates the overlap of the identified voxels with each WM tract are listed in detail.

**Table 2:** **Anatomic regions of the GM components of interest**.

Area	Brodmann area	Vol. (cm^3^)	*Z* max value (L/R) (*x*, *y*, *z*)
**GM IC6 (JOINT)**			
Positive			
Superior temporal gyrus	13, 22, 38, 39, 41	4.4/3.4	3.6 (−48, −40, 8)/4.6 (48, −38, 7)
Middle temporal gyrus	21, 22, 37, 39	5.4/1.3	4.5 (−48, −35, 2)/3.5 (48, −32, 2)
Middle frontal gyrus	6, 8, 9, 46	3.4/1.5	3.7 (−50, 16, 32)/3.0 (50, 19, 32)
Inferior frontal gyrus	9, 44, 45, 47	3.8/0.1	3.1 (−50, 10, 33)/2.1 (42, 30, 12)
Negative			
Middle temporal gyrus	21	0.7/0.3	3.1 (−45, −55, 6)/2.6 (42, −52, 8)
Parahippocampal gyrus	30	0.3/0.2	3.0 (−24, −46, 5)/2.6 (27, −46, 5)
**GM IC7 (JOINT)**			
Positive			
Superior temporal gyrus	21, 22, 39	1.0/2.0	2.9 (−48, −40, 8)/3.6 (50, −26, −1)
Middle temporal gyrus	19, 20, 21, 22, 39	1.8/2.9	3.2 (−48, −32, 2)/3.5 (48, −26, −4)
Inferior frontal gyrus	13, 46	1.2/1.6	2.7 (−39, 30, 12)/3.1 (39, 35, 9)
Parahippocampal gyrus	28, 36	1.3/1.0	2.8 (−27, −12, −15)/2.4 (30, −7, −17)
Fusiform gyrus	37	0.8/0.4	2.8 (−48, −47, −13)/2.5 (48, −47, −13)
Negative			
Precentral gyrus	4, 6	6.1/6.0	4.3 (−24, −23, 65)/3.3 (15, −23, 67)
Lingual gyrus	18	0.6/1.0	4.0 (3, −73, −6)/4.2 (12, −82, −14)
Paracentral lobule	4, 5, 6, 31	2.6/2.5	4.2 (0, −29, 51)/3.9 (3, −32, 51)
Postcentral gyrus	1, 2, 3, 5, 7, 40	4.3/3.3	4.1 (−21, −26, 65)/3.0 (50, −29, 51)
Medial frontal gyrus	6, 8, 32	3.0/4.2	4.1 (0, −23, 56)/3.6 (3, −20, 56)
Posterior cingulate	29	0.3/0.4	3.2 (−3, −58, 6)/3.6 (3, −58, 6)
Superior frontal gyrus	6, 8	3.4/3.2	3.3 (0, 5, 49)/3.2 (21, −8, 67)
Precuneus	7, 39	1.4/4.9	3.3 (−30, −62, 34)/3.2 (9, −74, 42)
Inferior parietal lobule	40	1.6/2.0	3.3 (−42, −35, 54)/3.3 (48, −32, 54)
**GM IC4**			
Positive			
Middle temporal gyrus	19, 21, 22, 37, 39	6.2/2.2	3.7 (−42, −69, 15)/2.9 (53, −58, 11)
Superior temporal gyrus	13, 22, 38, 39, 41, 42	5.2/2.6	3.5 (−53, −57, 19)/3.0 (50, −52, 14)
Supramarginal gyrus	40	2.9/2.4	3.4 (−53, −54, 22)/2.8 (53, −45, 30)
Precuneus	7, 19, 23, 31, 39	3.2/6.0	3.2 (0, −51, 36)/3.3 (3, −36, 43)
Parahippocampal gyrus	19, 28, 34	2.3/0.9	3.2 (−24, −38, 5)/2.7 (24, −41, 5)
Cingulate gyrus	24, 31, 32	2.0/2.1	3.1 (0, −42, 35)/3.2 (3, −33, 40)
Anterior cingulate	25	0.6/0.3	3.1 (0, 5, −8)/2.7 (3, 5, −10)
Postcentral gyrus	2, 40	2.0/0.2	3.1 (−50, −33, 49)/2.1 (50, −32, 51)
**GM IC5**			
Positive			
Precuneus	7, 19, 39	2.9/1.5	4.0 (−24, −65, 36)/4.6 (30, −59, 36)
Cerebellum		8.8/7.8	3.7 (0, −47, −8)/3.5 (3, −50, −8)
Middle frontal gyrus	6, 10	1.0/0.7	3.6 (−33, 39, 20)/2.9 (33, 47, 6)
Thalamus		1.8/1.0	3.5 (−6, −23, 12)/2.7 (3, −14, 12)
Middle temporal gyrus	19, 21, 22, 37, 39	1.8/0.9	3.1 (−48, −38, 5)/2.9 (48, −35, 2)
Negative			
Superior temporal gyrus	21, 38	1.5/0.6	3.1 (−30, 16, −24)/2.4 (45, 20, −16)
**GM IC9**			
Positive			
Superior temporal gyrus	22, 38	1.4/2.5	3.1 (−45, 11, −11)/3.7 (48, 11, −6)
Cuneus	7, 17, 18, 23, 30	2.6/0.7	3.5 (−12, −93, 5)/2.4 (18, −96, 8)
Superior frontal gyrus	6, 8, 9, 10	4.0/3.1	3.3 (−24, 48, 31)/3.1 (21, 11, 49)
Middle frontal gyrus	6, 8, 9, 10	5.3/2.6	3.1 (−33, 58, 3)/2.7 (27, 3, 52)
Precuneus	7, 19, 31	1.5/0.6	3.1 (−27, −62, 34)/2.9 (30, −62, 36)
Medial frontal gyrus	6, 8, 10, 32	1.3/1.1	3.1 (0, 11, 44)/3.0 (21, 5, 49)
Negative			
Middle temporal gyrus	19, 22, 39	1.8/1.5	3.9 (−48, −43, 5)/5.0 (42, −57, 22)
**GM IC10**			
Positive			
Angular gyrus	39	0.6/0.4	3.7 (−33, −54, 36)/3.8 (36, −56, 36)
Precuneus	7, 19, 39	1.5/0.6	3.7 (−30, −62, 36)/3.1 (36, −62, 36)
Supramarginal gyrus	40	0.4/0.4	3.1 (−36, −51, 36)/3.2 (36, −51, 36)
Middle frontal gyrus	6, 8, 9, 10	1.0/2.6	3.0 (−33, 16, 27)/2.9 (33, 19, 27)
Lingual gyrus	17	1.7/0.5	3.0 (−12, −87, 2)/2.6 (18, −87, 4)
Negative			
Inferior frontal gyrus	9, 44, 45, 47	2.7/2.1	3.7 (−48, 14, −3)/3.7 (48, 17, −6)
Superior temporal gyrus	22, 38, 42	4.2/1.7	3.7 (−48, 11, −6)/3.2 (50, 14, −6)
Insula	13	1.6/0.1	3.5 (−45, 8, −5)/2.2 (45, 8, −5)

**Table 3:** **White-matter tract labels of the FA components of interest**.

Abbreviation	WM tracts	Vol. (cm^3^)	%	*Z* max (R/L)
**FA IC7 (JOINT)**				
Positive				
ATR	Anterior thalamic radiation	2.3/7.2	5/14	4.7 (26, 31, 13)/5.2 (28, 25, 6)
CST	Corticospinal tract	2.1/2.3	6/7	5(25, 33, 7)/5.1(31, 34, 14)
CG	Cingulum	0.5/0.7	2/2	2.9(18, 21, 18)/3.1(28, 14, 31)
FM	Forceps minor/Forceps major	1.7/3.4	3/7	3.9(27, 47, 21)/5(27, 26, 22)
IFO	Inferior fronto-occipital fasciculus	1.1/2	2/5	3.9(16, 11, 22)/3.7(35, 45, 21)
ILF	Inferior longitudinal fasciculus	1.7/3.1	4/7	3.9(12, 19, 17)/5.3(41, 31, 15)
SLF	Superior longitudinal fasciculus	5.6/4.6	5/4	4.8(6, 25, 15)/5.4(44, 27, 15)
UF	Uncinate fasciculus	0.3/0.5	3/4	3.8(22, 51, 13)/2.9(40, 37, 10)
Negative				
ATR	Anterior thalamic radiation	1.1/0.9	2/2	3.3(20, 38, 27)/3.4(27, 27, 4)
CST	Corticospinal tract	1.9/1.4	5/4	3.5(25, 27, 7)/4.6(29, 31, 8)
SLF	Superior longitudinal fasciculus	3.2/4.1	3/4	5.2(12, 39, 29)/6(46, 30, 11)
**FA IC4**				
Positive				
ATR	Anterior thalamic radiation	0.8/4.2	2/8	7.8(27, 26, 2)/7.4(28, 24, 1)
CST	Corticospinal tract	2.7/1.9	7/6	8.5(26, 26, 1)/9.3(27, 26, 1)
ILF	Inferior longitudinal fasciculus	0.7/2.2	2/5	2.9(11, 32, 12)/4.2(44, 30, 12)
SLF	Superior longitudinal fasciculus	1.6/3.0	2/3	5.6(4, 26, 17)/5.3(48, 29, 10)
Negative				
ATR	Anterior thalamic radiation	2.3/1.2	6/4	4.2(24, 24, 8)/4.3(28, 31, 11)
IFO	Inferior fronto-occipital fasciculus	2.1/1.7	4/4	3.6(19, 9,23)/3.7(40, 15, 25)
ILF	Inferior longitudinal fasciculus	2.1/1.4	5/3	3.4(13, 15, 18)/3.3(45, 32, 13)
SLF	Superior longitudinal fasciculus	4.4/6.3	5/6	5(7, 27, 15)/5.1(48, 29, 14)

**Table 4:** **Anatomic regions of the group-discriminating fMRI components**.

Area	Brodmann area	Vol. (cm^3^)	*Z* max value (L/R) (*x*, *y*, *z*)
**ALFF – IC 6 (JOINT)**			
Positive			
Superior frontal gyrus	8, 9, 10, 11	3.8/4.8	9.5 (−30, 43, −15)/9.5 (21, 43, −17)
Middle frontal gyrus	6, 10, 11, 46, 47	6.5/5.8	7.9 (−30, 40, −17)/7.8 (30, 40, −17)
Inferior frontal gyrus	11, 46, 47	2.4/3.3	7.4 (−24, 31, −19)/6.0 (15, 31, −17)
Medial frontal gyrus	10, 11, 25	5.8/6.8	6.0 (−12, 28, −17)/6.1 (9, 43, −17)
Superior temporal gyrus	22, 38	0.4/0.4	3.5 (−56, 11, −6)/2.8 (59, 11, −6)
Anterior cingulate	10, 25, 32	1.0/0.3	3.5 (−12, 49, −5)/2.3 (15, 46, −5)
Thalamus		0.3/0.2	3.0 (−6, −11, 14)/3.0 (6, −5, 11)
**ALFF – IC 7 (JOINT)**			
Positive			
Superior frontal gyrus	6, 10, 11	0.8/0.3	5.4 (−18, 64, 8)/3.4 (9, 67, 8)
Superior temporal gyrus	22, 38	5.4/0.1	4.9 (−33, 13, −28)/2.3 (30, 10, −31)
Medial frontal gyrus	10	0.9/0.0	4.3 (−6, 64, 5)/−999.0 (0, 0, 0)
Inferior frontal gyrus	44, 45, 46, 47	2.0/0.0	4.0 (−53, 20, −9)/−999.0 (0, 0, 0)
Middle frontal gyrus	10, 11	1.3/0.3	3.4 (−42, 52, −10)/3.7 (30, 62, 19)
Negative			
Cingulate gyrus	23, 24, 32	2.2/2.8	3.5 (−9, 4, 27)/4.1 (9, 4, 27)
Anterior cingulate	24, 33	0.5/0.8	3.5 (−6, 10, 24)/4.0 (12, 13, 24)
Superior frontal gyrus	8, 10, 11	1.1/1.7	3.7 (−30, 32, 51)/3.9 (18, 43, −15)
Middle temporal gyrus	21, 38, 39	0.1/0.8	3.0 (−56, −66, 28)/2.6 (62, −35, −8)
**ALFF – IC 3**			
Positive			
Superior frontal gyrus	6, 8, 9, 10, 11	14.3/14.1	6.6 (−21, 57, 28)/6.5 (18, 65, 16)
Middle frontal gyrus	6, 8, 9, 10, 11, 46	12.5/11.6	5.0 (−27, 59, 19)/5.6 (24, 62, 19)
Medial frontal gyrus	6, 8, 9, 10	4.9/4.5	4.7 (−3, 49, 42)/4.7 (3, 49, 42)
Inferior frontal gyrus	9, 10, 45, 46, 47	2.6/2.0	3.9 (−42, 55, 0)/2.9 (56, 10, 33)
Superior temporal gyrus	38	0.6/0.1	3.1 (−42, 19, −26)/2.2 (39, 22, −26)

### Classification based on selected components

After transferring the group-discriminating components into *Z* values and thresholded at |*Z*| > 3.5, the mask from each component were generated and applied to the raw input matrix of each modality, resulting in three feature matrices in dimension of subject by voxels, i.e., FA: 63 × 312, ALFF 63 × 566, GM 63 × 1035, which served as the input to the further classification based on uni-modal and multimodal features.

**Figure 4:**
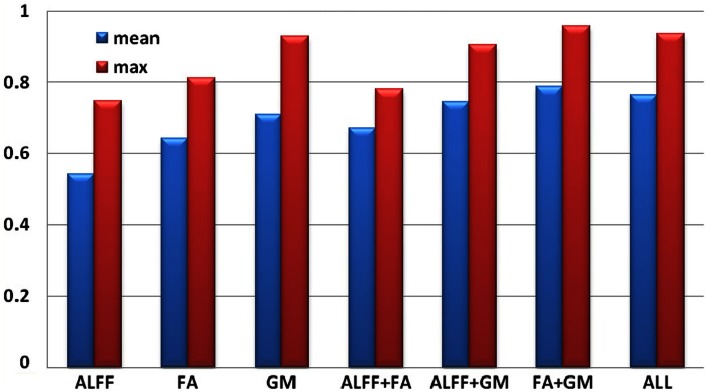
**Classification accuracy based on selected group-discriminative components from mCCA + jICA for seven modal combinations**.

Each individual was assigned one of two class memberships (SZ versus HC) and we have seven modal combinations (three single, three pair-wise, one three-way) as shown in Figure 4. After comparison, RSVM achieved the best classification accuracy among the four algorithms we trained with each of seven modal combinations; its mean, and maximum rates were summarized in Figure 4, where GM features obtained the highest accuracy in single modality, while FA + GM predict best among all seven modal combinations (mean 0.79, max 0.96).

## Discussion

In this paper we applied the mCCA + jICA model to three-way fusion of resting state fMRI, sMRI, and DTI data. The aim of the method is to identify precise correspondence among *n* data types and make possible the investigation of both shared and distinct abnormalities spanning multiple modalities for a specified brain disorder. Some abnormalities may occur in specific modalities, while others may be found in more than one modality simultaneously. Also, hidden linkages between components from different modalities may underlie in the data.

### Group differences

IC 7 significantly differentiated SZ from HC in all three modalities, suggesting the following abnormalities in SZ: (a) prefrontal cortex and left superior temporal gyrus (STG) (rest fMRI); (b) ATR, corticospinal tract (CSF), and forceps major (FMAJ; WM, DTI); and (c) regions of the motor cortex, medial/superior frontal cortex, and temporal gyrus (GM density). Furthermore, these identified affected regions may share some underlying relationship in SZ. The FA changes in ATR, CST, and FMAJ were previously associated with disconnectivity of brain networks in SZ in separate studies (Schlosser et al., 2007; Friedman et al., 2008; Sussmann et al., 2009). In particular, ATR projects from the anterior and medial regions of the thalamus to the frontal lobe, while CST subserves motor control. Accordingly, GM_IC7 shows strong alterations in motor cortex and, corresponding nicely to findings in Douaud et al. (2007) where the abnormalities in the primary sensorimotor and premotor cortices and in WM CST tracts were detected. Moreover, ALFF_IC7 implicates prefrontal cortex as abnormal, which plays an important role in the sensory integration and has been frequently reported dysfunction in SZ (Badcock et al., 2005; Hamilton et al., 2009; Yu et al., 2012a). These two pairs of components (FA-ALFF IC7, FA-GM IC7) depict a set of functional-anatomical “connected” regions. Note that both pairs have significant correlations (0.31/0.38) between their subject-mixing profiles as mentioned before, suggesting that disrupted WM connectivity may contribute to coordinated brain dysfunction, especially in the frontal and motor cortex, which is frequently hypothesized to be “disconnected” from other brain regions in SZ (Williams et al., 2004). Our results suggest that the anisotropy changes may relate to functional/structural changes in brain connectivity that are thought to play a central role in the clinical expression of SZ (Douaud et al., 2007).

Furthermore, GM-ALFF IC6 is another joint group-discriminative component, with middle/medial frontal cortex and thalamus (Woodward et al., 2012) indicated in ALFF map and temporal/frontal cortex shown in GM changes. The abnormality in each component have been previously found associated with the SZ deficits separately (Onitsuka et al., 2004; Zhou et al., 2007a; Edgar et al., 2012). Specifically, the result in Jayakumar et al. (2005) was in well accordance with our findings that SZ patients have significantly smaller global and regional GM volumes in inferior frontal, superior temporal, and parahippocampal gyri etc. Our results also suggest that functional disconnectivity associated with frontal lobe (also shown in ALFF_IC3) is present in SZ during rest (Hoptman et al., 2009). This is consistent with the notion that deregulation of medial frontal regions is associated with self-directed thoughts. This may lead to confusion between the source of internal and external stimuli, and may provide a neurophysiological basis for hallucinations (Whitfield-Gabrieli et al., 2009). This would have to be verified in future work.

We also identified ICs of interest showing significance only in one modality, such as GM_IC 5, 9, 10 and ALFF_IC3 (pink frame). The three structural components indicated regions including STG, precuneus, prefrontal cortex, insula, and thalamus, Hence, GM concentrations were significantly reduced in the above regions in the SZ group, consistent with other findings (Ha et al., 2004; Chua et al., 2007; Segall et al., 2009). Since structurally segregated and functionally specialized regions of the human cerebral cortex are interconnected by a dense network of cortico-cortical pathways (Hagmann et al., 2008; Segall et al., 2012), supporting the hypothesis that the SZ deficit may lie in aberrant structural changes and disconnectivity among different cortical areas.

### Correlation with positive symptoms

Positive symptoms refer to an excess or distortion of normal psychological functions, e.g., hallucinations and delusions. In Figure 3, the higher positive symptoms were correlated with identified voxels in the middle/STG, precuneus, anterior cingulate, and the parahippocampal gyrus in GM_IC4. This is consistent with similar findings in fMRI (Garrity et al., 2007) where PANSS positive scores were associated with abnormal activation of STG, precuneus. Similarly GM volumes in anterior and posterior cingulate regions were correlated with positive symptoms (Choi et al., 2005; Yan et al., 2012). Additionally, in Meda et al. (2012), similar regions were also reported in resting state fMRI data, in which anterior default mode and frontal-occipital regions have significant correlation with the PANSS positive subscale in SZ. All these findings suggest a general hypothesis that psychotic symptoms derive from functionally disconnected brain circuits, e.g., the disintegrated brain connectivity between medial frontal/prefrontal and parietal networks in SZ (Zhou et al., 2007b). For FA_IC4, the FA values in left ATR and SLF showed a significant negative correlation with positive PANSS, consistent with (Caprihan et al., 2008; Cui et al., 2011), suggesting that deficits of WM integrity in left frontal-parietal lobe may also be involved in the pathophysiology of positive symptoms. Finally, this data also supports the hypothesis that the failure of left-hemisphere lateralization might be involved in the pathophysiology of SZ (Szeszko et al., 2005).

### Classification based on selected ICs

The classification in Figure 4 shows that GM feature achieves the best classification among three single modalities, consistent with the fact that the selected GM components have much smaller *p* values than ALFF or FA. The most powerful prediction can be accomplished by using features from FA + GM, which is able to detail the multifaceted pathology that is likely to be present in SZ compared with single modality. Our results suggest that multimodal fusion of the selected group-discriminative components can improve the potential diagnosis prediction, in accordance with Sui et al. (2009a) and Yang et al. (2010), however, fusing as many modalities as possible in the training sample does not guarantee best classification rates, as we showed here and reported in Zhang et al. (2012); thus it would be helpful to compare a combination of uni-modal and multimodal results, as we did in Kim et al. (2010), to detect the potential biomarkers. We plan to pursue this possibility in future work by using larger data sets and various modalities, which aims to have bigger effect size and achieve higher accuracy.

### Future work

In this paper we develop and evaluate a novel multivariate method that can explore cross-information in multiple (more than two) data types and applied it to compare SZ patients to controls using an fMRI-DTI-sMRI combination. This is a novel attempt to perform a fusion of three different imaging modalities. The method described here could be applied straightforwardly to study other brain diseases (or subsets of a particular illness, such as psychotic or non-psychotic bipolar disorder). In addition, the choice of which multimodal data type to utilize is flexible, i.e., EEG, MEG, or genetic data, different features like fractional ALFF (fALFF) from fMRI (Kalcher et al., 2013) are also applicable. In a recent study, we found both ALFF and fALFF to be interesting and decided to start with ALFF (Turner et al., 2012), and will consider fALFF in future work. Finally the proposed method is very computationally efficient.

A limitation to the current study is that the subject number is not very high. Several statistical tests did not survive from the multiple comparisons, which may be complemented in future studies by including more subject samples or by multi-site recruitment. Additionally, mCCA + jICA operates on extracted features, rather than the original imaging data (e.g., using FA values instead of raw DTI data). Although some of the information is lost using this method, a “feature” tends to be more tractable than working with the large-scale original data due to the reduced number of dimensions (Calhoun and Adali, 2009) and provides a simpler space to link the data (Smith et al., 2009). Note that in our study we did not perform WM tractography but provided a type of summary statistic. A major strength of mCCA + jICA is that it can discover changes in one modality, e.g., which are related to alterations in distant, but connected regions in other modalities, without requiring a direct link.

Another point worth noting is that we did not collect physiologic data during the rest fMRI session as studies of patients tend to make this more difficult to collect. However it would be worth evaluating this in future work. With the advent of more rapid scanning (e.g., multiband sequences) which can adequately sample the cardiac noise, it is becoming much more feasible to characterize physiologic noise in large patient studies. We did not collect information on nicotine use either in these subjects, which may have potential effects on the imaging results, and would better be taken into account in the future. For example, recent studies indicated evidences of smoking effect in resting-state networks (Janes et al., 2012) and more prevalence in subjects with psychiatric disorder like SZ (Dickerson et al., 2013).

Multimodal fusion is an effective approach for analyzing biomedical imaging data that combines multiple data types in a joint analysis. It helps to identify the unique and shared variance associated with each imaging modality that underlies cognitive functioning in HCs and impairment in mental illness. In this real-world fusion application, we highlighted data from rest fMRI, WM tract, and GM concentration from SZ and healthy control subjects. We identified both modality-common and modality-unique group-discriminating aspects that verified the abnormalities in SZ, as well as replicated and extended previous findings. Such observations add to our understanding of the neural correlates of SZ. The proposed model promises a widespread utilization in the neuroimaging community and may be used to identify potential brain illness biomarkers.

## Conflict of Interest Statement

The authors declare that the research was conducted in the absence of any commercial or financial relationships that could be construed as a potential conflict of interest.

## Footnotes

^1^http://cobre.mrn.org

^2^http://www.fil.ion.ucl.ac.uk/spm/software/spm8

^3^www.fmrib.ox.ac.uk/fsl
